# The Enhanced Growth Performance and Antioxidant Capacity of Juvenile *Procambarus clarkii* Fed with Microbial Antioxidants

**DOI:** 10.3390/antiox14020135

**Published:** 2025-01-23

**Authors:** Zeyi Cheng, Jie Shi, Chen Qian, Jinghao Li, Xugan Wu, Ieong Kong, Jiayao Li

**Affiliations:** 1 Key Laboratory of Integrated Rice-Fish Farming Ecosystem, Ministry of Agriculture and Rural Affairs, Shanghai Ocean University, Shanghai 201306, China; m230150468@st.shou.edu.cn (Z.C.); shijie199767@163.com (J.S.); qianchenjoy@163.com (C.Q.); yxhdyhtc@163.com (J.L.); 2 National Demonstration Center for Experimental Fisheries Science Education, Shanghai Ocean University, Shanghai 201306, China; xgwu@shou.edu.cn; 3 Shanghai Engineering Research Center of Aquaculture, Shanghai Ocean University, Shanghai 201306, China; 4 Key Laboratory of Freshwater Aquatic Genetic Resources, Ministry of Agriculture and Rural Affairs, Shanghai Ocean University, Shanghai 201306, China; 5 Shanghai Chuangbo Ecological Engineering, Shanghai 201108, China; kongieong1988@gmail.com

**Keywords:** microbial antioxidant, growth performance, antioxidant capacity, resistance to air exposure stress, *Procambarus clarkii*

## Abstract

Given the economic significance of *Procambarus clarkii* in freshwater aquaculture and the lack of microbial antioxidants in *Procambarus clarkii* diet research, this study aimed to investigate the optimal supplementation level and feeding duration of microbial antioxidants in *Procambarus clarkii* diets. A series of three experiments were conducted to assess the long-term effects of different MA levels on crayfish and evaluate the palatability of the diets by observing feeding behavior and examining the short-term effects of high levels of MA. Our results indicate that long-term feeding using 1.5% MAs markedly increased the activities of antioxidant enzymes (T-AOC, T-SOD, and GSH-PX) and decreased the malondialdehyde (MDA) content in the hepatopancreas and hemolymph, with the crayfish showing significantly higher survival rates due to better antioxidant capacity after 24 h of air exposure stress. Under the condition of long-term feeding, the appropriate level of addition of MAs that can promote the growth of crayfish is 0.62–0.66%. The feeding behavior results indicate that the lower willingness and food intake of the crayfish in the high MA group may be the main reason affecting their growth. Conversely, short-term feeding using MAs alleviated the adverse effects on growth associated with the reduced palatability of the diet. The results indicate that the inclusion of 1.5% MAs in the diet for a period of 21 d optimized crayfish growth, accompanied by an improvement in antioxidant capacity and survival during transportation. This study demonstrates that diets supplemented with microbial antioxidants (MAs) can improve growth performance, antioxidant capacity, and resistance to air exposure stress in *Procambarus clarkii*. These results provide valuable insights into the potential benefits of MA supplementation in crayfish aquaculture.

## 1. Introduction

The red swamp crayfish *Procambarus clarkii* is one of the most significant freshwater crustaceans in China [1,2]. It is a popular consumer item, due to its rapid growth, adaptability, high nutritional value, and delicious taste [3,4]. At present, the annual yield of China’s crayfish has reached 3,161,022 tons [5]. The primary method of crayfish transportation is via off-water means, which subjects crayfish to the dual stressors of dehydration and hypoxia. This disrupts their respiratory metabolism, prompting the body to produce a substantial quantity of reactive oxygen species (ROS) [6]. The ROS inflict oxidative damage to the tissues and diminish the survival rate if antioxidant enzymes cannot be promptly eliminated, resulting in greater economic losses [7]. Therefore, improving the health status of juvenile crayfish and reducing oxidative stress during transport is essential for the sustainable development of aquaculture [8,9].

In recent years, the use of probiotics and their metabolites in aquaculture to protect the health of farmed animals has become a trend [10,11]; various probiotic micro-organisms have been used as feed additives in aquaculture with some positive results [12,13], where *yeasts*, *Bacillus*, *Lactobacillus*, *Micrococcus*, *Enterococcus*, and *Lactobacillus* are widely used as probiotics in animal husbandry and aquaculture [8,12,14,15]. Microbial antioxidants (MAs) are novel compounds fermented by probiotics that are known to promote growth and improve nutrient absorption, as well as feed utilization and antioxidant capacity [16,17]. They can also replenish vitamins and amino acids. Furthermore, they facilitate the production of a range of active substances during the metabolic process, including proteases, amylases, and lipases. These substances enhance the digestion and absorption of nutrients, thereby improving the feed conversion ratio (FCR) and ensuring the healthy growth of aquatic animals [18,19]. A recent study reported that the dietary addition of MA increased lipase activity, promoted growth performance, and enhanced the immunity of *Eriocheir sinensis* by promoting antioxidant capacity against the negative effects of unfavorable environmental factors [20].

It can be reasonably proposed that the utilization of MAs as a dietary supplement for crayfish with the objective of enhancing its antioxidant capacity represents a viable solution to the issue of oxidative stress during the transportation process. This has the potential to significantly enhance the overall benefits of crayfish farming. MAs, as a feed additive, can maximize the improvement of the physiological functions of farmed animals and reduce the cost of farming, and are widely used in livestock diets. Numerous studies of mammals have found that the addition of MAs to diets can improve the growth performance in *Sus scrofa domestica* [17], *mus musculus* [16], and *gallus domestics* [14], but currently, there are few applications of MAs in aquatic animals [20]. Therefore, it is important to determine the performance of crayfish and the optimal MA supplementation level and feeding duration. The aim of this study was to investigate the effects of MAs as a feed additive on the growth performance and antioxidant capacity of crayfish, and to provide a theoretical basis for the use of MAs as a feed additive for crayfish, as well as a new way to alleviate oxidative stress during the transport of crayfish.

## 2. Materials and Methods

### 2.1. Experimental Diets

The experimental feeds used in this study were made by adding different doses of microbial antioxidants (MAs) to a commercial compound feed (Wuhan Dabeinong group, Wuhan, China) for crayfish. The MAs were made from the fruits of sea buckthorn and Rosa roxburghe, which were fermented by beneficial microbes, such as *Bacillus subtilis*, *Lactobacillus*, and *Saccharomyces,* through a compound fermentation process (The main components were as follows: *Bacillus subtilis* ≥ 1.0 × 10^10^ cfu/g, *Lactobacillus* ≥ 1.0 × 10^7^ cfu/g, and *Saccharomyces* ≥ 1.0 × 10^7^ cfu/g). It is then extracted, concentrated, inactivated, lyophilized, and subjected to other processes, and it contains carotenoids, vitamins B1, B2, B12, and C, Quercetin-3-D-glucopyranose (quercetin), Quercetin (flavonoids), inositol, and metal derivatives of various trace elements (Shanghai Chuangbo Ecological Engineering, Shanghai, China). For the specific antioxidant content of the antioxidant components, see the study on microbial antioxidant on antioxidant performance and immune function in mice [21]. The diet preparation procedure was as follows: the pelleted diets were recrushed and sieved through a 60-mesh sieve with the addition of MAs at levels of 0% (A1), 0.25% (A2), 0.5% (A3), 1% (A4), and 1.5% (A5). To reduce the loss of vitamins due to comminution, the diets of each group were supplemented with 1% vitamin premix, weighed according to the ratio, and all ingredients were thoroughly mixed and then made into a 2 mm particle size diet by adding an appropriate amount of distilled water. The diets were dried and stored at -40 °C and protected from light. The experimental diets were analyzed for their nutrient composition according to standard procedures [22,23]. The crude protein (34%, dry matter) and crude fat (8.7%, dry matter) contents of the experimental diets were able to meet the growth requirements of juvenile crayfish [24]. The specific dietary ingredients and nutrient composition are shown in Table 1.

### 2.2. Experimental Design

#### 2.2.1. Trial 1: Different Levels of Microbial Antioxidant Feed Experiment

The experiment was conducted in September 2022, and the crayfish used in this study were vigorous juvenile crayfish with healthy appendages, selected from the Chongming base of Shanghai Ocean University. They were kept in an indoor recirculating water monoculture system containing dechlorinated tap water (temperature: 24.0 ± 1 °C; pH: 8.0 ± 0.5; dissolved oxygen: >7 mg/L; total ammonia nitrogen: below 0.2 mg/L; nitrite content: below 0.01 mg/L), and were fed commercial diets for 7 d of acclimatization. Subsequently, 240 crayfish of a similar size (initial average weight: 3.95 ± 0.6 g) were selected and randomly divided into five groups, with 48 crayfish in each group. The crayfish were fed the A1–A5 diets at 3–5% of their body weight daily, at 18:00, during the feeding trial. The following morning, every day of the experiment, all tanks were cleaned by siphoning out feed residues and feces to maintain water quality. Crayfish molting, water temperature, and dissolved oxygen were recorded daily; water quality indicators were measured twice a week, and the water was changed or added as appropriate, according to water quality. At the end of the 70 d feeding trial, the crayfish were fasted for 24 h. The surface water of the crayfish was then dried with absorbent paper, weighed with an electronic balance (accuracy of 0.01 g), and measured for body length and width, using a vernier caliper (accuracy of 0.01 mm). The hepatopancreas and hemolymph of 18 crayfish from each group were collected for the weighing and determination of oxidase activity.

After the feeding trial, 15 juveniles from each dietary treatment were randomly selected, removed from their tanks, and maintained in individual plastic boxes (8.5 cm × 6.5 cm × 12 cm; volume = 450 mL). The temperature and relative humidity of the room were maintained at 21 ± 0.7 °C and 39.25 ± 0.89%, respectively. After 24 h, all the surviving crayfish in each group were counted and sampled for subsequent determination of respiratory metabolic enzymes.

#### 2.2.2. Trial 2: Behavioral Experiments with Microbial Antioxidant Diets

The feeding behavior experiments were conducted in round polyethylene barrels (diameter: 50 cm), and a fixed infrared camera recording system (Hikvision, DS-2DC24021W-DE3) was placed directly above each drum to record the feeding behavior of the crayfish during the experimental process. Considering the behavior of crayfish in darkness, the behavioral observation was carried out from 18:00 to 21:00, and each crayfish was used only once during the whole experiment. The water and crayfish were replaced after the completion of one shot to start a new round of feeding behavior observation.

The temporary culture conditions for 1 week were the same as above, and then 52 juvenile crayfish of a similar size (initial average weight: 7.04 ± 0.43 g) were divided into four groups that were fed with only A1 and A5 diets under satiation or starvation (for 48 h), with 10 replicates for each of the two feeding groups. Due to the small size and limited food intake of 3 g of juvenile crayfish, which also tend to become satiated more quickly, 7 g of juvenile crayfish with a longer feeding time were used in the feeding behavior trial. One crayfish was placed in the experimental barrel and allowed to acclimatize to the water environment for 2 h. The 20 experimental diets were weighed and placed in the PC tube in the center of the barrel, and the crayfish were fixed in the PC tube area directly in front of the diet to ensure that the crayfish in each group had the same distance of access to the diet. At the same time, the two PC tubes were removed, so that the feeding behavior of the crayfish could be automatically recorded for 3 h. After the experiment, the residual feed was immediately collected, dried at 65 °C for 24 h, and weighed.

#### 2.2.3. Trial 3: Short-Term Optimization Experiments with Microbial Antioxidant Diets

The temporary culture conditions for 1 week were the same as above, and then 240 crayfish of a similar size (initial average weight: 5.62 ± 0.85 g) were selected and randomly divided into two groups, control group A1 (0% microbial source antioxidant) and antioxidant group A5 (1.5% microbial source antioxidant), with 150 crayfish in each group, and three replicates were set up. The experimental management was as described above. The experiment was carried out for 28 d. After feeding for 7 d, 14 d, 21 d, and 28 d, 18 crayfish were collected from each of the two feeding groups for growth statistics and antioxidant enzyme activity measurements (the methods used were the same as Trial 1), and 18 crayfish were randomly selected from each of the two feeding groups for 24 h air exposure stress experiments under the same conditions as above. After 24 h, all the surviving crayfish in each group were counted and sampled for the subsequent determination of respiratory metabolic enzymes.

### 2.3. Index Determination

The weight growth rate (WGR), specific growth rate (SGR), survival rate (SR), meat yield (MY), and hepatosomatic index (HSI) were calculated according to the following formulas:

WGR (%) = (W_t_ − W_0_)/W_0_ × 100 (1)

SGR (%/d) = (ln W_t_ − lnW_0_)/D *×* 100 (2)

SR (%) = N_f_/N_i_
*×* 100 (3)

MY (%) = W_m_/W_t_
*×* 100 (4)

HSI (%) = W_h_/W_t_
*×* 100 (5)

where W_0_ is the initial weight of the crayfish (g), W_t_ is the final weight of the crayfish (g), and D is the number of experimental days. N_f_ is the final crayfish numbers, and N_i_ is the initial crayfish numbers. W_m_ is the muscle weight (g), and W_h_ is the hepatopancreas weight (g).

For the determination of conventional nutrients, the moisture content was determined by drying the samples at 105 °C, using a constant weight method. The crude protein content was quantified by digesting the samples with concentrated sulfuric acid and then by employing the Kjeldahl method (TM 8200, Foss, Hoganas, Sweden). The total lipid content was determined using the Folch method [22], which employs a chloroform–methanol solution and a solution of 0.37 mol/L KCl for extraction. The samples were subjected to a muffle furnace (PCDE3000, Shanghai, China) analysis to determine the ash content in accordance with the AOAC methodology [23].

The hemolymph and hepatopancreas of 18 crayfish from each group after Trial 1 and Trial 3 were thawed, weighed, and homogenized, separately, in nine volumes (*v*/*v*) of a precooled physiological saline solution, using a T10B homogenizer (IKA Co., Staufen, Germany). The homogenates were centrifuged at 10,000× *g* for 10 min at 4 °C, and the supernatants were collected using a pipette for later analysis. Subsequently, the total superoxide dismutase (T-SOD), total antioxidant capacity (T-AOC), glutathione peroxidase (GSH-PX), and malondialdehyde (MDA) levels were detected in the supernatants of the hepatopancreas and hemolymph after Trial 1 and Trial 3. The hepatopancreas and muscle of six crayfish from each group after air exposure stress were thawed, and hepatopancreas treatment was the same as above. Muscle tissue was crushed and homogenized, separately, in nine volumes (*v*/*v*) of a precooled physiological saline solution, using a T10B homogenizer. Subsequently, MDA, lactic acid (LD), lactic dehydrogenase (LDH), and succinate dehydrogenase (SDH) levels were analyzed in the supernatants of the hepatopancreas and muscle after air exposure stress. All the above parameters were determined using the corresponding bio-kits (Nanjing Jiancheng Bioengineering Institute, Nanjing, China) with a T6 New Century spectrophotometer (Beijing Purkinje General Instrument Co., Ltd., Beijing, China), according to the manufacturer’s instructions.

We watched and analyzed the behavior observation videos, recorded using an infrared monitoring system, and calculated the duration of various behaviors [25]. The statistical behavior parameters mainly included the following:

Dissolution rate (%) = 100 *×* (Initial feed weight − dried feed weight)/initial feed weight (6)

Food intake (g) = Initial feed weight − dried feed weight/(1 − dissolution rate)(7)

Searching time (min): Time of first exposure of crayfish to feed(8)

Feeding time (min): Time for crayfish to sniff, test, and ingest feed(9)

Willingness to feed (s): Time to start feed ingestion − time of first feed exposure(10)

where the initial feed weight is the weight of the feed that is not put into the water, and the dried feed weight is the weight of the feed taken out of the water after 3 h and dried.

### 2.4. Statistical Analysis

SPSS 22.0 software (SPSS, Michigan Avenue, Chicago, IL, USA) was used for statistical analysis. Levene’s equal variance test and the Shapiro–Wilk test were used to test for the homogeneity of variance and normal distribution of all data, respectively. Under the same feeding period, one-way analysis of variance (ANOVA) was used to analyze whether there was a significant difference between crayfish that were fed different MA levels. If the one-way ANOVA was significant, a Duncan’s range test was used to further analyze the significance of the different treatments. A Student’s *t*-test was used to analyze the growth property differences between the control and MA treatments for short-term feeding, and it was also used to determine the significant differences between the behavioral indicators of crayfish in A1 and A5 in the same state of starvation or satiation. The significance level was set at *p* < 0.05. The data are presented as mean ± standard deviation (SD). Based on the actual amount of MAs in the diet, regression analysis was used to determine the optimal level of MA supplementation, using a quadratic curve model (Supplementary Table S1).

## 3. Results

### 3.1. Growth Performance and Whole-Body Proximate Composition

**Figure 1:**
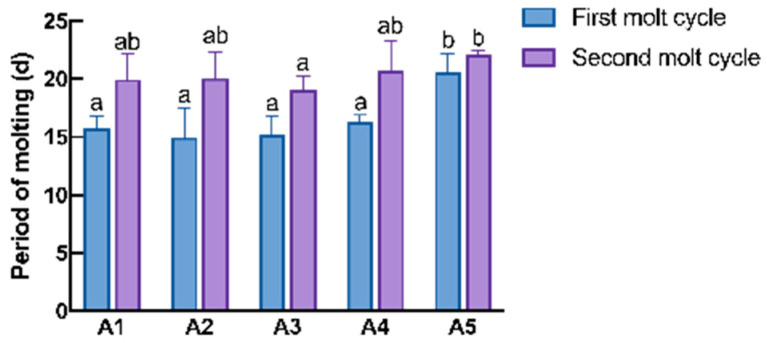
Effects of different levels of microbial antioxidants in the diet on the molting cycle of crayfish. Note: The data are presented as mean ± SD (*n* = 48). The superscripts in the figure indicate a significant difference (*p* < 0.05).

**Figure 2:**
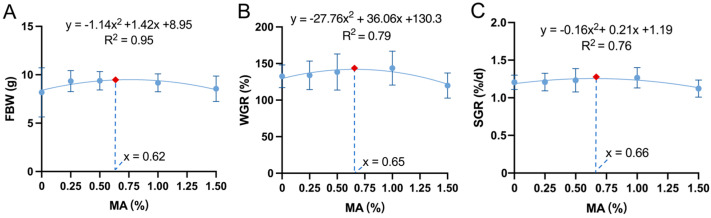
Quadratic relationship between dietary microbial antioxidant supplementation and the FBW (**A**), WGR (**B**), and SGR (**C**) of juvenile crayfish (*n* = 33).

After 70 d of feeding using the A1–A5 group diets, the SR of juvenile crayfish in the diet group with added MAs was significantly higher than that of the A1 group, and with an increase in MAs in the diet, FBW, WGR, and SGR showed a trend of increasing and then decreasing, with the highest being in the A4 group, the second highest in the A3 group, and the lowest in the A5 group (*p* < 0.05; Table 2); the MY was highest in the A4–A5 groups, and the HSI was highest in the A5 group. There was no significant difference in the final length and width of juvenile crayfish between groups (*p* > 0.05; Table 2). In addition, the crude protein content in muscle was highest in group A4, and significantly lower in group A5 than in groups A1–A4 (*p* < 0.05); the ash content in muscle was lower in groups A4–A5 than in groups A1–A3 (*p* < 0.05; Table 3). The A5 group was significantly longer than the other groups in both molt cycles, and A3 was significantly shorter than the other groups in the second molt cycle (*p* < 0.05; Figure 1). A regression analysis of the FBW, WGR, and SGR on the MA level was used to determine the optimal level of MA supplementation, using a quadratic curve model. With increasing MA concentrations, the FBE, WGR, and SGR all showed a trend of increasing and then decreasing, to peak at 0.62%, 0.65%, and 0.66%, respectively (Figure 2).

### 3.2. Antioxidative Capacity

**Figure 3:**
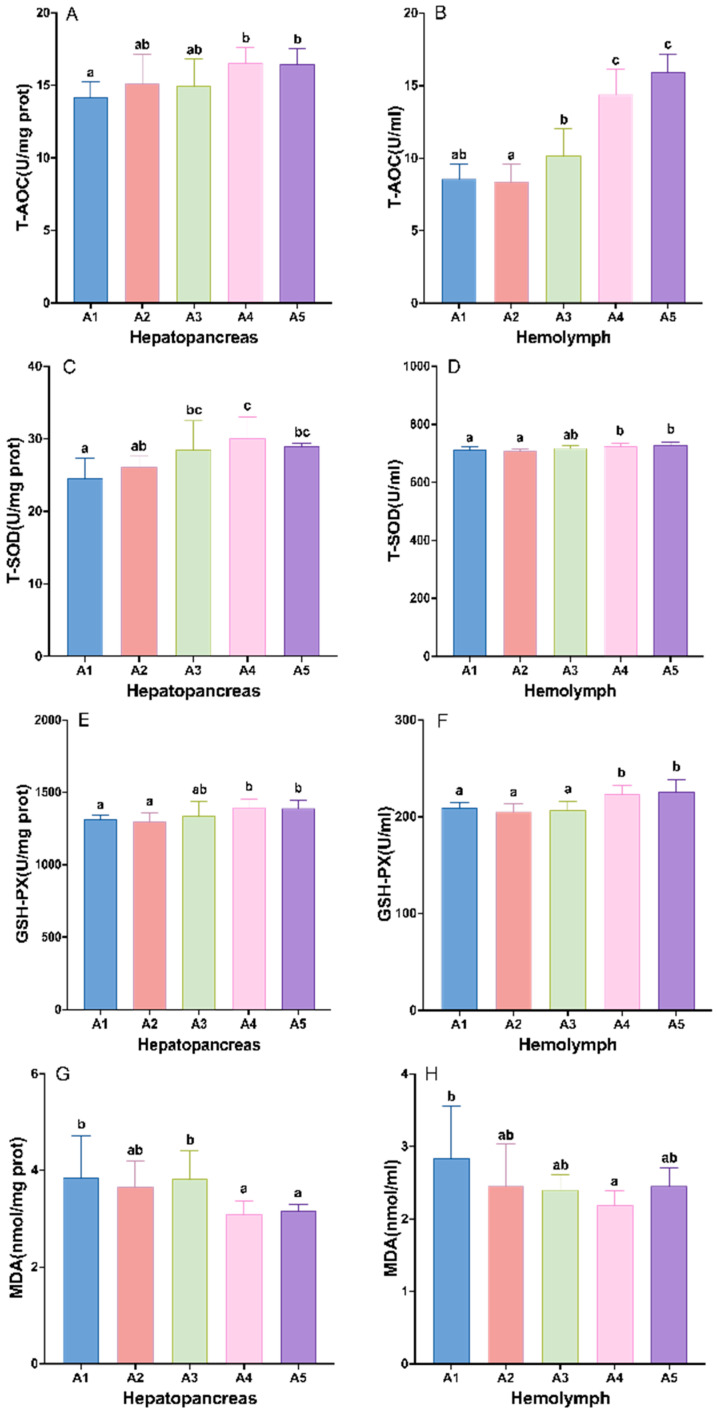
Effects of different levels of microbial antioxidants on hepatopancreas and hemolymph antioxidant enzyme activities in terms of T-AOC (**A**,**B**), T-SOD (**C**,**D**), GSH-PX (**E**,**F**), and MDA (**G**,**H**) content in juvenile crayfish. Note: The data are presented as mean ± SD (*n* = 6). The different letters on the top of the bars mean significant differences (*p* < 0.05).

The parameters related to the antioxidant capacity of crayfish were significantly affected by the level of antioxidant supplementation from MAs, and the addition of antioxidants from MAs to the diet significantly increased the activities of T-AOC, T-SOD, and GSH-PX, and decreased the content of MDA in the hepatopancreas and hemolymph of juvenile crayfish (*p* < 0.05). In the hepatopancreas, the activities of T-AOC and GSH-PX showed a trend of increasing with increasing MA doses, T-AOC and GSH-PX activities were significantly higher in groups A4–A5 than in group A1 (*p* < 0.05; Figure 3A,E), and the MDA content was significantly lower than in group A1 (*p* < 0.05; Figure 3G). The activities of T-SOD were significantly higher in groups A3–A5 than in group A1, and peaked in group A4 (*p* < 0.05; Figure 3C). In the hemolymph, the contents of T-AOC, T-SOD, and GSH-PX showed an increasing trend with an increase in MA supplementation, and were all significantly higher in groups A4–A5 than in groups A1–A3 (*p* < 0.05; Figure 3B,D,F); the MDA contents of juvenile crayfish in the MA-supplemented groups were lower than in the control group, and showed a decreasing and then increasing trend, reaching the lowest level in group A4 (*p* < 0.05; Figure 3H).

### 3.3. Stress Response to Air Exposure

**Figure 4:**
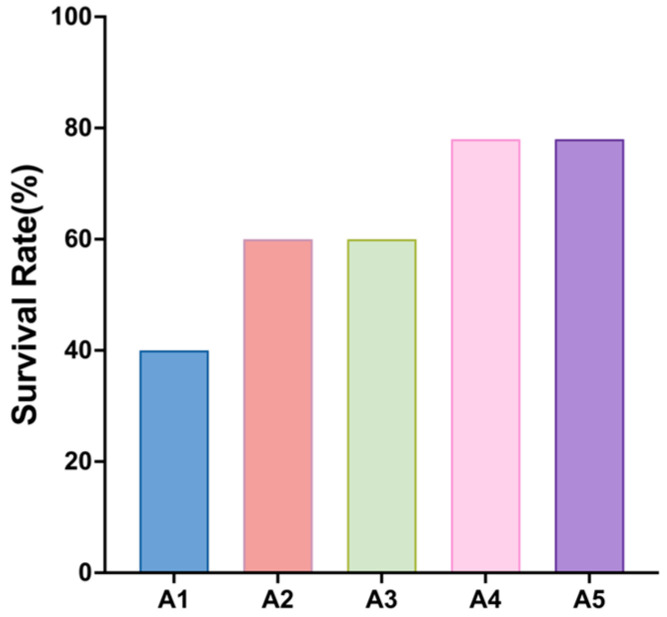
Effects of different levels of microbial antioxidants on the survival rate of juvenile crayfish after 24 h of air exposure stress.

After 24 h of air exposure stress, the survival rate of the crayfish in each group gradually increased with increasing addition of MA to the diet, and the survival rate of juvenile crayfish in the MA-added group was significantly higher than that of the control group, with the highest survival rates in groups A4 and A5 (Figure 4).

**Figure 5:**
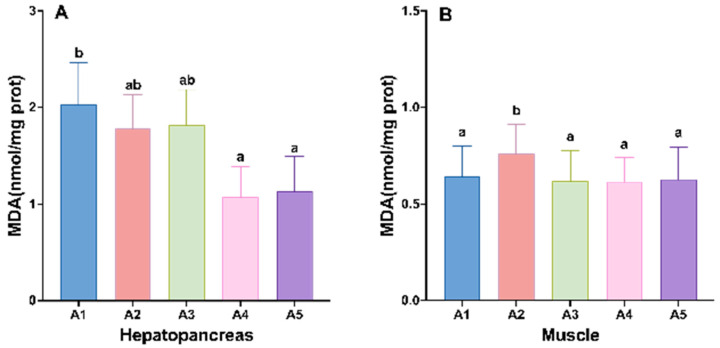

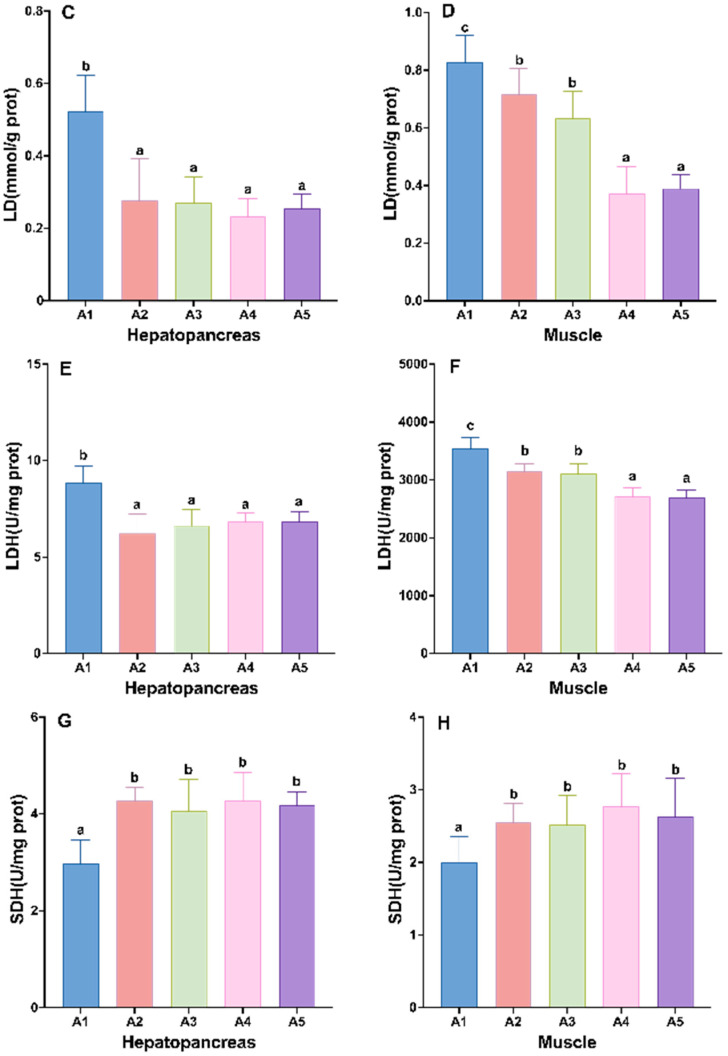
Effects of different levels of microbial antioxidants on the hepatopancreas and muscle in terms of the MDA (**A**,**B**) content and LD (**C**,**D**), LDH (**E**,**F**), and SDH (**G**,**H**) activities in crayfish after air exposure stress. Note: The data are presented as mean ± SD (*n* = 6). The different letters on the top of the bars mean significant differences (*p* < 0.05).

The parameters related to the antioxidant capacity of juvenile crayfish exposed to air stress were significantly affected by the level of MA supplementation, and the addition of MAs to the diet significantly decreased LD content and LDH activity in the hepatopancreas and muscle of juvenile crayfish (*p* < 0.05). In the hepatopancreas, the MDA, LD, and LDH contents of juvenile crayfish in the MA-supplemented group were significantly lower than those in the control group, with the MDA content being lowest in the A4 and A5 groups, and significantly lower than that in the A1 group (*p* < 0.05; Figure 5A); LD content and LDH activity were highest in the A1 group and significantly lower in the A2–A5 groups, with no significant difference between groups (*p* < 0.05; Figure 5C,E); and SDH activity was significantly higher in the A2–A5 groups than in the A1 group (*p* < 0.05; Figure 5G). In the muscles, both LD content and LDH activity were significantly lower in groups A2–A5 than in group A1 (*p* < 0.05; Figure 5D,F), and SDH activity was significantly higher in groups A2–A5 than in group A1, with no significant difference between groups A2–A5 (*p* > 0.05; Figure 5H); MDA content among the groups was not significantly different between the A1 group and the A3–A5 groups (*p* > 0.05; Figure 5B).

### 3.4. MA Feed Palatability

In the starvation condition, the time spent searching for food (5.6 min) and the willingness to feed (0.3 s) in group A1 was shorter than that in group A5 (8.4 min and 0.8 s), but there was no significant difference after the analysis of the results (*p* > 0.05, Table 4); In the satiation condition, the time spent searching for food (12. 9 min) in group A1 was significantly shorter than that in group A5 (23.2 min) (*p* < 0.05, Table 4). In the satiation condition, the time interval between exposure to A1 and the start of eating (0.7 s) was significantly shorter than in group A5 (4.8 s) (*p* < 0.05, Table 4), so the crayfish’s willingness to feed on A5 was significantly weaker than that on A1.

When the crayfish were in the starved state, there was no significant difference between the feeding time of group A1 and group A5 (*p* > 0.05; Table 4); when in the satiated state, the feeding time of crayfish in group A5 (24.8 min) was significantly longer than that of the crayfish in group A1 (16.5 min) (*p* < 0.05; Table 4). During the 3 h of filming, whether the crayfish were starved or satiated, the intake of group A1 (0.18 g and 0.17 g) was higher than that of group A5 (0.13 g and 0.10 g) (*p* < 0.05; Table 4). In general, the crayfish ingested significantly less of the MA-supplemented diet than the unsupplemented group in both the starvation and satiation conditions, and the crayfish had a lower willingness to ingest the MA-supplemented diet for a longer period in the satiation condition.

### 3.5. Short-Term Feeding Optimization

The growth performance of the crayfish in groups A1 and A5 showed an increasing trend for FBW and WGR with increasing feeding time (*p* < 0.05; Table 5), which reached the highest at 14 d, but there was no significant difference in terms of WGR and SGR between the two groups, according to the growth performance results at 7, 14, and 21 d of feeding (*p* > 0.05). The SGR shows an increasing and then decreasing trend for the juvenile crayfish in group A5, which was significantly lower than that in group A1 (*p* < 0.05); there was no significant difference in HSI and MY between the two groups at the four feeding periods (*p* > 0.05; Table 5).

**Figure 6:**
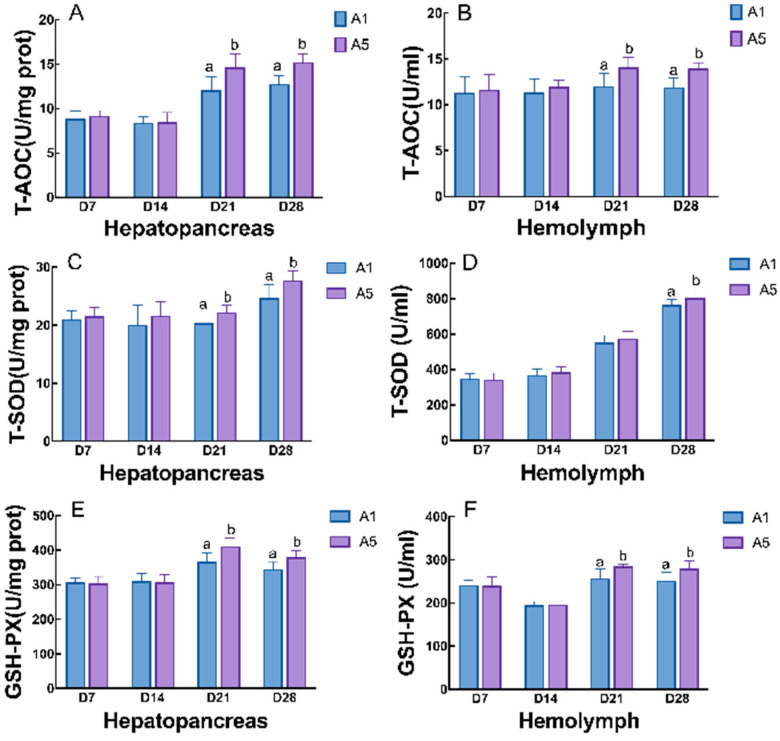

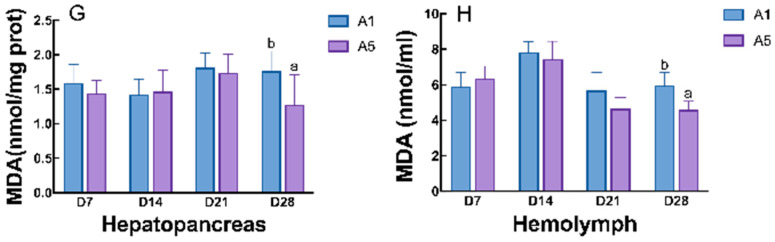
Effects of different microbial antioxidant feeding times on the antioxidant parameters in the hepatopancreas and hemolymph of juvenile crayfish, in terms of T-AOC (**A**,**B**), T-SOD (**C**,**D**), GSH-PX (**E**,**F**), and MDA (**G**,**H**) content. Note: The data are presented as mean ± SD (*n* = 6). The different letters on the top of the bars mean significant differences (*p* < 0.05).

The parameters related to the antioxidant capacity of juvenile crayfish were significantly affected by the duration of MA feeding, as the activities of T-AOC, T-SOD, and GSH-PX in the hepatopancreas and hemolymph of the juvenile crayfish increased with increasing duration of MA feeding, while the content of MDA decreased (*p* < 0.05; Figure 6). In the hepatopancreas, the activities of T-SOD, T-AOC, and GSH-PX in group A5 were found to be significantly higher than those in group A1 after 21 d and 28 d of feeding, reaching their peak at 28 d of feeding (*p* < 0.05; Figure 6A,C,E); the MDA content in group A5 showed a tendency to increase and then decrease, with the lowest antioxidant content at 28 d of feeding, which was significantly lower than group A1 (*p* < 0.05; Figure 6G). In the hemolymph, the activities of T-AOC and GSH-PX in the A5 group were significantly higher than those in the control group after 21 d and 28 d of feeding, reaching a peak at 21 d (*p* < 0.05; Figure 6B,F). The MDA content of the juvenile crayfish at 28 d of feeding was significantly lower than that of the A1 group (*p* < 0.05; Figure 6H). In the hepatopancreas and hemolymph, the indicators of the four antioxidant enzymes of the crayfish in the two groups at the time periods of 7 d and 14 d of feeding did not show any statistical differences (*p* > 0.05).

**Figure 7:**
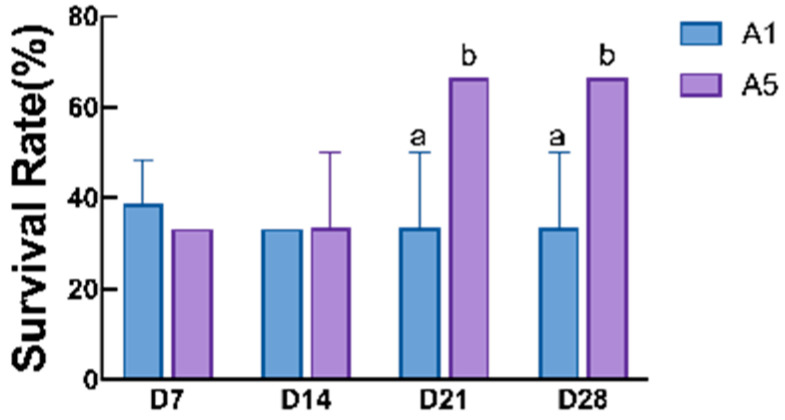
Effects of different microbial antioxidant feeding times on the survival rate of juvenile crayfish after 24 h of air exposure stress. The different letters on the top of the bars mean significant differences (*p* < 0.05).

**Figure 8:**
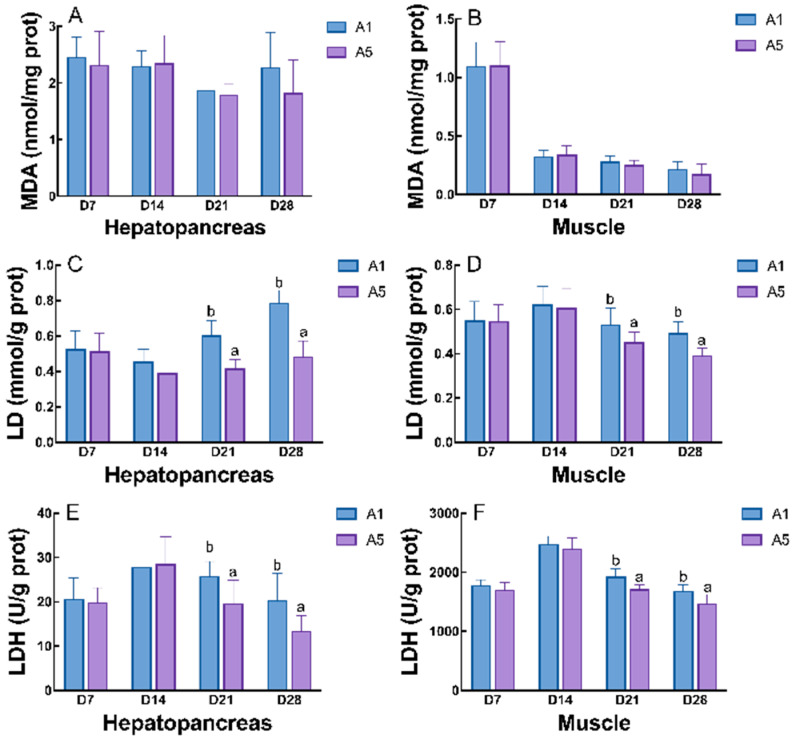

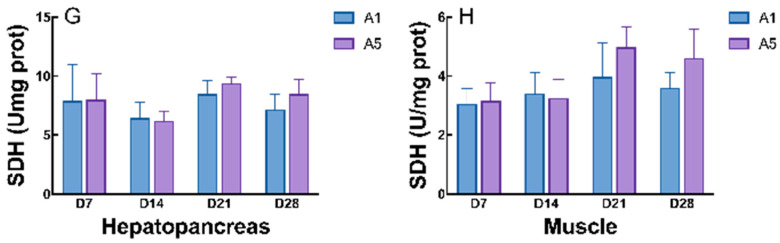
Effects of different microbial antioxidant feeding times on the hepatopancreas and muscle of crayfish, in terms of MDA (**A**,**B**) content and LD (**C**,**D**), LDH (**E**,**F**), and SDH (**G**,**H**) activities, after 24 h of air exposure stress. The data are presented as mean ± SD (*n* = 6). The different letters on the top of the bars mean significant differences (*p* < 0.05).

After 24 h of air exposure stress, the crayfish in the A5 group showed the highest survival rates after 21 d and 28 d of feeding, which were significantly higher than that of the control group (*p* < 0.05; Figure 7). The parameters related to the antioxidant capacity of air exposure stress in the juvenile crayfish were significantly affected by the antioxidant feeding period, and the addition of MAs to the diet both significantly reduced LD content and LDH activity in the hepatopancreas and muscle of the crayfish (*p* < 0.05; Figure 8). In the hepatopancreas, the MDA, LD, and LHD contents of the crayfish in group A5 were significantly lower than those in group A1, with the MDA content of the crayfish at 28 d of feeding being significantly lower than that of group A1 (*p* < 0.05; Figure 8A). The LD content and LDH activity of group A5 were significantly lower than those of group A1 at 21 d and 28 d of feeding (*p* < 0.05; Figure 8C,E); the SDH content of group A5 was higher than that of group A1 after 21 d and 28 d of feeding (*p* < 0.05; Figure 8G). In the muscles, the LD content and LDH activity began to show a decreasing trend after 14 d of feeding, and were significantly lower in group A5 than in group A1, at 21 d and 28 d (*p* < 0.05; Figure 8D,F); the SDH activity increased significantly at 21 d and 28 d of feeding, and it was significantly higher in group A5 than in group A1, reaching a peak at 21 d (*p* < 0.05; Figure 8H); and there was no significant difference between the two groups for the MDA content (*p* > 0.05; Figure 8B).

## 4. Discussion

The components of microbial antioxidant additives have been demonstrated to enhance the ecological balance of intestinal flora, facilitate growth and development, prevent and control diseases, and improve production performance [19]. Previous studies on mammals found that daily weight gain was significantly increased by the addition of 0.5% MAs fed to piglets for 2 weeks [26] and 0.8% MAs fed to mice for 3 weeks [27]. In a study on crustaceans, the WG and SGR of *E. sinensis* were significantly higher than those of the control group after 56 d of adding 0.2% microbe-derived antioxidant feed [20]. In the present study, it was found that the addition of 0.25–1% MAs to the diet under long-term feeding significantly increased the weight gain rate of crayfish; however, the addition of 1% MAs could make the WGR and SGR of juvenile crayfish significantly higher than those of the other groups, and the content of crude protein and crude fat in the body was also higher than that of the other groups. The MAs include components such as VC, and numerous studies have shown that feed supplements of VC can significantly improve specific growth rates and reduce mortality in crustaceans [28,29]. The results of studies utilizing microbial-fermented feeds in aquatic animals have also demonstrated that growth performance can be enhanced. Panigrahi et al. [30] found that probiotic bacteria, such as *B. subtilis,* in bio-flocculent systems significantly increased *Penaeus indicus* WGR, MY, and FCR, and improved survival. Wang et al. [31] found that *Macrobrachium nipponense* consuming probiotic-supplemented diets containing *Lactobacillus* improved survival and optimized growth. It can thus be concluded that the fermentation components and metabolic yields of MAs can facilitate crayfish growth.

Studies on functional feed additives have highlighted that overdosing can lead to adverse effects, with dosage being a crucial factor influencing probiotic efficacy [32]. Several studies have demonstrated that excessive probiotic supplementation in diets impairs the growth performance of aquatic animals [19,33]. Zhang et al. [34] reported limited, or even negative, effects on the growth and physiological functions of crustaceans when astaxanthin was overdosed, and similarly, excess VC in the diet reduced weight gain in crayfish, compared to the control group [29]. Appropriate taurine levels (10 and 25 mg kg^−1^) accelerated molting and improved survival in juvenile *L. vannamei* [35], whereas excessive taurine (>0.8%) negatively impacted growth, nutrient accumulation, and survival in *E. sinensis* [36]. In terms of MA fermentation components, sea buckthorn fermentation produces *γ-aminobutyric acid* (GABA), which is also present in the fermentation products of *Clostridium butyricum*, and may affect the effectiveness of the diet due to its content. Meng et al. [37] found that high concentrations of *C. butyricum* supplements (2%) inhibited growth in *Micropterus salmoides*, while low concentrations (0.5%) enhanced body weight and total fatty acid content in the gut and liver, positively modulating intestinal flora. In this study, the 1.5% MA group exhibited the longest molting cycles and reduced the final body weight (FBW), weight gain rate (WGR), and specific growth rate (SGR). Conversely, the 0.5% MA group had the shortest second molting cycle, aligning with regression analyses indicating optimal additions for growth performance. Therefore, the incorporation of microbial antioxidants in diets should adhere to the principle of moderation. Based on the conditions of this study, 0.65% MA feed is suitable for long-term feeding in order to optimize the growth performance of crayfish.

Although the addition of 1.5% MAs affected growth performance, the antioxidant capacity indices in both the hepatopancreas and hemolymph of juvenile crayfish increased with an increase in MAs in the diet, and the best antioxidant capacity was found in the crayfish that were fed an addition of 1–1.5% MAs. Gu et al. [38] also found that mice being gavaged with 1% MAs could significantly increase the vitality of GSH-PX and SOD in the hemolymph and reduce the MDA content at the same time so that the antioxidant defense system in the body was enhanced and the antioxidant capacity of the mice was improved. Shi et al. [19] also found that, when *E. sinensis* ingested diets supplemented with 0.2% MAs, T-AOC and T-SOD were significantly higher than that of the control group, and MDA was significantly reduced under ammonia nitrogen stress. This is due to the presence of *B. subtilis*, *Lactobacillus*, and *flavonoids* in MAs, which can improve the antioxidant capacity of juvenile crayfish. *B. subtilis* can effectively inhibit the formation of lipid peroxidation products [39]. Prasuna et al. [40] found that adding *B. subtilis* to the diet could increase the SOD and CAT activities in the blood of *Macrobrachium rosenbergii*, reduce the MDA content, and improve antioxidant capacity. The same results were found in *Penaeus vannamei* regarding ingesting fermented feed supplemented with *B. subtilis* [39]. *Lactobacillus* can produce SOD in the organism, which is also an important barrier against free radical attack [41]. In crayfish, Li et al. [2] showed that T-AOC and SOD activities significantly increased and MDA content reduced after the intake of probiotic bio-continues containing *Lactobacillus*, *Bacillus,* and other probiotic organisms, which improved the immune and antioxidant capacity of the crayfish. The same results were found in *Fennerpenaeus chinensis* [42]. *Flavonoids* can reduce lipid peroxidation and ROS, and play a role in lymphocyte protection [43]. Li et al. [44] found that, when crayfish ingested diets supplemented with different concentrations of fermented *Moringa oleifera* leaves (*flavonoids*, etc.), this increased the hepatopancreatic SOD and GSH-PX activities and reduced the MDA content compared to the basal diet, with the addition of 1% fermented *M. oleifera* leaves being the most effective in improving antioxidant capacity. The present study showed that different levels of MAs enhanced the antioxidant defense system in crayfish, and the addition of 1–1.5% MAs was the best for the antioxidant capacity of juvenile crayfish.

The dual stress of dehydration and hypoxia during transport disrupts respiratory metabolism, induces oxidative damage to tissues, and reduces the survival of crustaceans [6,45]. Air exposure stress generates a large amount of ROS in the organism, which can cause oxidative damage if antioxidant enzymes are unable to remove them in a timely manner [46]. Respiratory metabolic enzyme results further confirmed that different levels of MAs could potentially mitigate the oxidative damage caused by air exposure stress on the organism. Antioxidant components, such as VC, *taurine*, and *isoflavones,* in MA products can eliminate ROS produced by the organism due to air exposure stress and reduce the degree of anaerobic respiration [47], which improves the resistance of crayfish to desiccation. *Flavonoids* can reduce lipid peroxidation and ROS, and play a role in lymphocyte protection [43]. In the hepatopancreas and muscle of crayfish, 1.5% MAs showed the lowest LD content and LDH capacity and the highest SDH capacity. These results further indicate that MAs can prolong the transition from aerobic to anaerobic respiration under air exposure stress [48]. Furthermore, the lowest levels of MDA, reflecting free radical metabolism and internal oxidative damage in the form of lipid oxidation end products [49], were found in the 1–1.5% MA groups. Dong et al. [36] found that, when taurine was added to the diet of *E. sinensis* at different concentrations, the MDA content in the plasma and hepatopancreas of *E. sinensis* was significantly lower in crabs fed the 0.4% and 0.8% *taurine* diets than in those fed the control diet. Thus, *taurine* may improve the response of crayfish to dehydration and hypoxia-induced stress during off-water transport. Thus, the antioxidant components in MA products reduced the response to dehydration and hypoxia-induced stress, and improved survival to air exposure stress. Although the 1.5% MA diet was effective in improving the antioxidant capacity and resistance to air exposure stress of crayfish in the long-term diet, the growth performance was also significantly lower than the other additive groups.

Feeding behavior is a basic living activity of animals, and the main way to obtain nutrients [25]. The weight gain of aquatic animals is closely related to dietary nutrients, and the palatability of the diet is also directly related to the growth and development of aquatic animals [50]. In terms of dietary nutrient composition, the crude protein, crude fat, and ash contents did not differ significantly among the additive groups, and there was no significant difference in body composition (Table 3) between the juvenile crayfish that ingested the 1.5% MA diet for 70 d and the other additive groups in terms of crude fat and ash contents. In the diet design, there was no difference in nutrient composition between the diet groups, but there was a dose difference in the addition of MAs. Therefore, the weight gain and palatability of crayfish were dependent on the level of MAs. From the perspective of feed palatability, a number of studies have shown that the addition of attractants influences the feeding behavior of aquatic animals [51,52]. Suresh et al. [53] found that the addition of small peptides and nucleotides significantly increased the intake of *Litopenaeus stylirostris*, which can be used as an attractant and palatability enhancer to influence the feeding behavior of animals, since MAs are produced by the fermentation of *Lactobacillus*, *B. subtilis*, and *yeast*. The fermentation of *Lactobacillus* produces organic acid (*lactic acid*) [54], which results in lower pH and higher acid concentration in fermented diets, exacerbating the sour taste of the diets [15]. The results of the feeding behavior showed that the 1.5% MA-supplemented group was significantly different from the control group in terms of seeking time, willingness to feed, feeding time, and feeding amount, and the amount of food intake by the crayfish was significantly lower than that of the control group in both the satiated and starved conditions; the willingness to feed was weaker, suggesting that the excessive addition of MAs had, indeed, affected the palatability of the diets. This finding is in accordance with the results of Zhang et al. [55]. When evaluating the sensory index of fermented feeds (fermented with different concentrations of *Lactobacillus*, *Brewer yeast*, and *B. subtilis*) for *P. vannamei*, it was found that the fermented feeds produced a sour taste compared to the unfermented diets. The crustaceans usually have a strong ability to recognize chemical information, including food odors [25]. Therefore, it can be reasonably inferred that the increase in sour taste brought about by adding more MAs to the feed may be the main reason affecting the palatability of crayfish feed. The low intake under long-term feeding reduced the nutrient accumulation of the juvenile crayfish in the 1.5% group, resulting in a prolonged molting period and lower growth performance than in other groups. Based on the positive effects of MAs on the growth and antioxidant capacity of crustacean aquatic animals, how to reduce the effect of MAs on feed palatability, and which attractant can improve the effect of MAs on palatability are also directions worthy of further research in the future.

Long-term feeding using the 1.5% MA diet has an effect on the growth performance of crayfish, as well as resulting in a significant improvement in the antioxidant capacity of the organism and resistance to air exposure stress. By optimizing the levels of MAs via feeding strategies to facilitate the better application of 1.5% MA supplements in crayfish production practice, using short-term feeding to determine the optimal feeding time for 1.5% MA supplements and improving resistance to air exposure stress with transport requirements can also be combined for cost reduction and efficiency. The short-term feeding results showed that the 1.5% MA diet had no negative effect on growth performance for 21 d (3 weeks), but after 28 d (4 weeks), the WGR and SGR of juvenile crayfish were significantly lower than those of the control group, indicating a negative effect on growth. The results were similar to those of *E. sinensis* fed on a diet supplemented with MAs, with a significant decrease in the weight gain rate after 28 d [19]. Some studies have shown that *lactic acid bacteria* and *yeast*-fermented diets are not suitable for long-term feeding [56,57]. Long-term feeding on *lactic acid bacteria* and *yeast* additive diets results in the continuous proliferation of live bacteria colonizing the gut [58], which can prey on and absorb nutrients in the gut of crayfish, affecting their growth performance. In terms of antioxidant capacity, the experimental results of Zhu [59], who added 1% MAs to the drinking water of rats, showed that the SOD and GSH-PX activities in serum and liver were significantly higher than those of the no-addition group after 28 d, and the MDA content reached the lowest level. The present study demonstrates that the application of 1.5% MAs enhances antioxidant capacity, with the activities of T-AOC and GSH-PX reaching a peak at 21 d, while the MDA content was also the lowest at 21 d. Therefore, feeding juvenile crayfish with a 1.5% MA diet for 21 d can enhance the antioxidant capacity and achieve the optimal antioxidant level. According to the indexes after air exposure stress, the survival rate of the 21 d and 28 d antioxidant groups was significantly higher than that of other groups, and the level of respiratory metabolic enzymes was also better than that of the other groups, indicating that the addition of 1.5% MAs to the diet for 21 d could reduce the degree of anaerobic respiration in the process of air exposure stress. Optimized feeding strategies may be a viable means of using high levels of MA-supplemented diets in crayfish production to meet transport requirements, reduce costs, and increase efficiency.

## 5. Conclusions

In conclusion, the addition of microbial antioxidants to a diet can improve the growth performance, antioxidant capacity, and immunity of juvenile crayfish, and reduce oxidative damage during transport. However, the addition of excessive amounts of microbial antioxidants to long-term diets was found to have limited beneficial effects on the growth and physiological functions of crayfish and negatively affect the palatability of the diets. By combining the indicators of growth, antioxidants, resistance to air exposure stress, and economic benefits, the addition of 1.5% microbial antioxidants to a diet for 21 d was effective in improving antioxidant capacity and reducing oxidative damage during transport.

## Figures and Tables

**Table 1:** Feed configuration and regular nutrient composition (dry matter).

Items	A1	A2	A3	A4	A5
Ingredients (%)					
Commercial feed	95	95	95	95	95
Vitamin premix	1	1	1	1	1
Carboxymethyl cellulose	2	2	2	2	2
Wheat meal	2	1.75	1.5	1	0.5
Microbial antioxidants ^1^	0	0.25	0.5	1	1.5
Proximate composition (%)					
Moisture	7.17	6.68	8.03	7.96	8.08
Crude protein	34.22	33.93	33.75	34.48	34.30
Crude lipid	8.81	8.92	8.72	8.99	8.88
Ash	16.40	17.17	17.43	15.79	16.90

^1^: MAs were composed of 503 mg/kg Fe, 367 mg/kg Mn, 1.07 mg/kg Cu, 0.18 mg/kg Se, 194,000 U/100 g SOD, 322 mg/100 g vitamin C, 908 µg/100 g vitamin E. 4.43% total flavones, 1.37% *isoflavones*, 886 mg/100 g glutathione, 82.4 mg/100 g total saponins, 4.21% total amino acids, and 0.146% *taurine*.

**Table 2:** Effects of different levels of microbial antioxidants (in feed) on survival, growth performance, and the hepatopancreas index of juvenile crayfish (*n* = 33).

Items	A1	A2	A3	A4	A5
IBW (g)	3.81 ± 0.56	4.01 ± 0.49	3.97 ± 0.49	3.79 ± 0.51	3.92 ± 0.71
FBW (g)	8.88 ± 1.24 ^ab^	9.35 ± 1.07 ^b^	9.38 ± 0.94 ^b^	9.16 ± 0.92 ^b^	8.55 ± 1.30 ^a^
WGR (%)	133.55 ± 15.46 ^b^	133.87 ± 19.18 ^b^	138.41 ± 24.22 ^b^	143.81 ± 22.88 ^b^	119.99 ± 17.02 ^a^
Final length (mm)	51.55 ± 2.31 ^a^	52.98 ± 1.99 ^bc^	53.70 ± 2.33 ^c^	53.19 ± 2.29 ^bc^	52.14 ± 2.52 ^ab^
Final width (mm)	15.05 ± 0.74	15.12 ± 0.66	15.00 ± 0.60	15.24 ± 0.57	15.05 ± 0.63
SR (%)	91.67 ± 5.89	97.92 ± 4.66	97.92 ± 4.66	97.92 ± 4.66	97.92 ± 4.66
SGR (%/d)	1.21 ± 0.09 ^b^	1.21 ± 0.11 ^b^	1.23 ± 0.15 ^b^	1.27 ± 0.13 ^b^	1.12 ± 0.11 ^a^
MY (%)	14.58 ± 1.98	14.63 ± 1.23	14.43 ± 2.04	15.38 ± 1.85	15.44 ± 2.18
HSI (%)	7.58 ± 1.01	7.65 ± 0.93	7.92 ± 0.98	7.76 ± 1.01	8.13 ± 1.17

Notes: The numerical values represent the mean and standard deviation (SD). The letters indicate significant differences between groups (*p* < 0.05).

**Table 3:** Effects of different levels of microbial antioxidants on the routine biochemical composition of juvenile crayfish muscle (wet mass: *n* = 6).

Items	A1	A2	A3	A4	A5
Moisture	76.97 ± 1.31	78.68 ± 1.97	78.19 ± 1.45	78.20 ± 1.92	77.52 ± 1.71
Crude protein	16.63 ± 2.09 ^ab^	17.05 ± 1.85 ^b^	16.88 ± 1.20 ^b^	17.39 ± 0.54 ^b^	14.87 ± 0.65 ^a^
Crude lipid	1.03 ± 0.15	0.89 ± 0.08	0.96 ± 0.09	0.98 ± 0.24	1.01 ± 0.11
Ash	3.07 ± 0.21	3.10 ± 0.31	3.06 ± 0.35	2.94 ± 0.32	2.98 ± 0.35

Notes: The numerical values represent the mean and standard deviation (SD). The letters indicate significant differences between groups (*p* < 0.05).

**Table 4:** Effects of different levels of microbial antioxidants on the behavioral parameters of juvenile crayfish (*n* = 10).

Items	Starvation		Satiation	
	A1	A5	A1	A5
Searching time (min)	5.6 ^a^	8.4 ^a^	12.9 ^a^	23.2 ^b^
Willingness to feed (s)	0.3 ^a^	0.8 ^a^	0.7 ^a^	4.8 ^b^
Feeding time (min)	16.8 ^a^	16.8 ^a^	16.5 ^a^	24.8 ^b^
Food intake (g)	0.18 ^b^	0.13 ^a^	0.17 ^b^	0.10 ^a^

Note: The numerical values represent the mean and standard deviation (SD). The letters indicate significant differences between groups (*p* < 0.05).

**Table 5:** Effects of the different feeding times of microbial antioxidant feed on the growth performance and hepatopancreas index of juvenile crayfish (*n* = 18).

Items	7 d		14 d		21 d		28 d	
	A1	A5	A1	A5	A1	A5	A1	A5
IBW (g)	5.51 ± 0.91	5.34 ± 0.73	5.08 ± 0.87	5.30 ± 0.94	5.57 ± 0.71	5.85 ± 0.83	5.45 ± 1.00	5.73 ± 0.98
FBW (g)	5.79 ± 0.95	5.65 ± 0.77	6.96 ± 1.22	7.20 ± 1.18	7.83 ± 1.07	8.16 ± 1.21	8.46 ± 1.55	8.50 ± 1.46
Final length (mm)	48.06 ± 2.36	46.86 ± 1.61	50.58 ± 2.10	50.71 ± 2.45	51.50 ± 2.17	51.81 ± 2.43	53.49 ± 2.28	53.05 ± 2.04
Final width (mm)	13.70 ± 0.91	13.73 ± 0.73	14.34 ± 0.61	14.28 ± 0.66	14.79 ± 0.75	15.04 ± 0.80	14.86 ± 1.05	14.85 ± 0.83
WGR (%)	5.04 ± 1.69	5.65 ± 1.42	36.92 ± 3.45	36.38 ± 5.70	40.41 ± 4.04	39.37 ± 3.82	55.09 ± 2.81 ^b^	48.34 ± 2.04 ^a^
SGR (%/d)	0.70 ± 0.23	0.78 ± 0.19	2.24 ± 0.18	2.21 ± 0.30	1.61 ± 0.14	1.58 ± 0.13	1.57 ± 0.06 ^b^	1.41 ± 0.05 ^a^
HSI (%)	7.08 ± 0.86	7.28 ± 0.96	6.86 ± 0.88	6.52 ± 0.76	6.67 ± 0.81	6.29 ± 0.68	7.13 ± 0.68	7.21 ± 0.86
MY (%)	16.53 ± 1.30	16.92 ± 1.66	14.38 ± 1.33	14.74 ± 1.61	12.86 ± 1.37	12.55 ± 1.42	15.06 ± 1.62	15.81 ± 2.30

Notes: The numerical values represent the mean and standard deviation (SD). The letters indicate significant differences between groups (*p* < 0.05).

## Data Availability

The data that support the findings of this study are available from the corresponding author upon reasonable request.

## Supplementary Materials

The following supporting information can be downloaded at: https://www.mdpi.com/article/10.3390/antiox14020135/s1, Supplement Table S1. Detailed data of quadratic relationship between dietary microbial antioxidant supplementation and FBW(A), WGR(B) and SGR(C) of juvenile crayfish.
