# Supplementary material for: The Enhanced Growth Performance and Antioxidant Capacity of Juvenile Procambarus clarkii Fed with Microbial Antioxidants
Source: Antioxidants (Basel). 2025 Jan 23;14(2):135. doi: 10.3390/antiox14020135 (PMC11851763; doi:10.3390/antiox14020135)
Supplement: Supplementary file 1 [file antioxidants-14-00135-s001.zip › antioxidants-3384793-supplementary.pdf]

Supplement Table S1. Detailed data of quadratic relationship between dietary microbial antioxidant supplementation and FBW(A)、WGR(B) and SGR(C) of juvenile crayfish.

FBW(g) Model Summary

R	R2	Adjusted R2	Error in standard estimation
0.975	0.950	0.942	0.078

Coefficient

	Non-standardized Coefficient		Standardization Coefficient	t	Significance
	B	Standard error	Beta		
VAR00001	1.420	0.141	2.446	10.040	0.000
VAR00001 ** 2	-1.139	0.090	-3.085	-12.665	0.000
constant	8.952	0.040		223.199	0.000

WGR (%) Model Summary

R	R2	Adjusted R2	Error in standard estimation
0.891	0.795	0.760	4.003

Coefficient

	Non-standardized Coefficient		Standardization Coefficient	t	Significance
	B	Standard error	Beta		
VAR00001	36.056	7.236	2.457	4.983	0.000
VAR00001 ** 2	-27.761	4.598	-2.977	-6.037	0.000
constant	130.269	2.052		63.497	0.000

SGR(%/d）Model Summary

R	R2	Adjusted R2	Error in standard estimation
0.873	0.763	0.723	0.027

Coefficient

	Non-standardized Coefficient		Standardization Coefficient	t	Significance
	B	Standard error	Beta		
VAR00001	0.210	0.048	2.296	4.331	0.001
VAR00001 ** 2	-0.165	0.031	-2.839	-5.357	0.000
constant	1.189	0.014		86.690	0.000
